# Exploring the Immediate Impact of *APOE* ε4ε4 Genotype Disclosure among Latinos

**DOI:** 10.1002/alz.086185

**Published:** 2025-01-03

**Authors:** Daniela Diaz Caro, Sophia Rodriguez, John Brandon Wetmore, Jonathan D Godinez, Itzel A Camarillo, Jill Goldman, Wendy R. Uhlmann, Sarah McCain, Maria Caban, Cheng‐Shiun Leu, Ana F Abraido‐Lanza, Rafael A. Lantigua, Wendy K Chung, J. Scott Roberts, Karolynn Siegel, Ruth Ottman

**Affiliations:** ^1^ Columbia University Irving Medical Center, New York, NY USA; ^2^ Taub Institute Columbia University Irving Medical Center, New York, NY USA; ^3^ University of Michigan Medical School, Ann Arbor, MI USA; ^4^ University of Michigan School of Public Health, Ann Arbor, MI USA; ^5^ Columbia University, New York, NY USA; ^6^ Boston Children’s Hospital, Boston, MA USA; ^7^ Harvard Medical School, Boston, MA USA; ^8^ New York State Psychiatric Institute, New York, NY USA

## Abstract

**Background:**

Alzheimer’s disease (AD) risk is markedly increased among *APOE* ε4/ε4 homozygotes. Previous studies of *APOE* genotype disclosure impact have included few ethnic minorities. This study addresses this gap by investigating the immediate impact of disclosing an *APOE* ε4/ε4 genotype in the Información de la Enfermedad de Alzheimer para Latinos (IDEAL) study, a Latino community‐based study.

**Methods:**

IDEAL participants attended semi‐structured genetic counseling sessions conducted via Zoom in Spanish or English by certified genetic counselors. Sessions included a PowerPoint presentation covering genetics, genotype results, risk estimates, and AD prevention methods. Risk estimates were based on data from the Washington Heights‐Inwood Columbia Aging Project, a longitudinal study conducted in the same communities. The estimate of risk to age 85 among ε4 homozygotes was 55%. We transcribed session recordings and conducted content analysis.

**Results:**

Eleven ε4/ε4 participants aged 41‐64 attended risk evaluation sessions (English 8, Spanish 3). Six had a family history of AD (first‐degree 1, distant 5). Nine were women and two men. Overall, participants did not show strong emotional reactions. Many expressed surprise, accompanied by thoughtful reflections on the connection between their AD risk and perceived memory problems or emotional well‐being. Participants generally appreciated the information provided during the genetic counseling sessions because it offered insights into the importance of AD risk awareness and prevention strategies.

**Conclusion:**

Latinos at high AD genetic risk showed limited immediate emotional response to risk disclosure and appreciated the information that was shared. Longitudinal analyses may provide further insight into the impacts of this disclosure.

This study is supported by the National Institute on Aging (R01 AG062528) and registered in ClinicalTrials.gov (NCT04471779).

**Keywords**: Alzheimer's disease, *APOE* ε4ε4, genetic counseling, Latinos, qualitative research, risk disclosure

